# Synthesis of Sr_2_Si_5_N_8:_Ce^3+^ phosphors for white LEDs via an efficient chemical deposition

**DOI:** 10.1038/srep45832

**Published:** 2017-03-31

**Authors:** Che-Yuan Yang, Sudipta Som, Subrata Das, Chung-Hsin Lu

**Affiliations:** 1 Department of Chemical Engineering, National Taiwan University, Taipei, Taiwan, ROC

## Abstract

Novel chemical vapor deposition (CVD) process was successfully developed for the growth of Sr_2_Si_5_N_8_:Ce^3+^ phosphors with elevated luminescent properties. Metallic strontium was used as a vapor source for producing Sr_3_N_2_ vapor to react with Si_3_N_4_ powder via a homogeneous gas-solid reaction. The phosphors prepared via the CVD process showed high crystallinity, homogeneous particle size ranging from 8 to 10 μm, and high luminescence properties. In contrast, the phosphors prepared via the conventional solid-state reaction process exhibited relative low crystallinity, non-uniform particle size in the range of 0.5–5 μm and relatively lower luminescent properties than the phosphors synthesized via the CVD process. Upon the blue light excitation, Sr_2−x_Ce_x_Si_5_N_8_ phosphors exhibited a broad yellow band. A red shift of the emission band from 535 to 556 nm was observed with the increment in the doping amount of Ce^3+^ ions from x = 0.02 to x = 0.10. The maximum emission was observed at x = 0.06, and the external and internal quantum efficiencies were calculated to be 51% and 71%, respectively. Furthermore, the CVD derived optimum Sr_1.94_Ce_0.06_Si_5_N_8_ phosphor exhibited sufficient thermal stability for blue-LEDs and the activation energy was calculated to be 0.33 eV. The results demonstrate a potential synthesis process for nitride phosphors suitable for light emitting diodes.

Presently, blue emitting InGaN based white light emitting diodes (WLEDs) are gathering enormous attention for indoor and outdoor lighting industries because of their elevated brightness, extended lifetime, and eco-friendliness^1^,^2^,^3^. One of the most famous phosphors for solid state lighting is yellow-emitting Y_3_Al_5_O_12_:Ce^3+^ phosphor, which can be efficient excited by the InGaN chip. However, WLEDs based on this phosphor have certain disadvantages including a poor color rendering index (CRI) at long wavelengths^2^,^3^. For enhancing the CRI of WLEDs, nitrodosilicate materials which are suitable for ultraviolet (UV)/blue InGaN chip have been studied significantly owing to their long-wavelength emissions and good thermal stability^4^,^5^. Nitridosilicate compounds possess covalent and short Re–N chemical bonds in condensed structures with corner-sharing or edge-sharing SiN_4_ tetrahedral frameworks^6^. Such compact structures provide strong crystal fields and covalent nature for nitridosilicate phosphors. The above characteristics result in a relatively broad excitation range, long emission wavelengths, and low thermal quenching behavior^7^,^8^,^9^,^10^,^11^.

Among the nitridosilicate phosphors, M_2_Si_5_N_8_: Eu^2+^ (M = Ca, Sr, Ba) are considered to be the significant orange-red phosphors for white LEDs (WLEDs) because of the high quantum efficiency under blue excitation and high thermal stability^12^,^13^. Ce^3+^-doped Sr_2_Si_5_N_8_ has been reported as one of the potential yellow phosphors for WLEDs^14^. However, the research on the detailed structural and photoluminescence analysis including quantum efficiency, concentration quenching and thermal quenching behavior are still few. For the fabrication of industrial Sr_2_Si_5_N_8_ phosphors, the conventional solid-state reaction process is known to be the most common approach^12^,^13^,^14^,^15^,^16^. However, the high reaction temperatures (1350–1600 °C) exceed the melting point for one of the constituent materials, Sr_3_N_2_ (m.p. = 1030 °C)^10^,^11^,^12^,^13^,^14^. Excessive liquid phase during the reaction causes non-homogeneous reactions, resulting in phosphors with low quality and insufficient emission intensity. Furthermore, owing to the air-sensitive properties of Sr_3_N_2_, the mixing process of raw materials needs to be carried out in a glove box^17^,^18^. The use of glove boxes is relatively complicated, thereby increasing the cost for preparing phosphors. Therefore, the innovation of a low cost approach that can be able for the mass production of high quality phosphors is desirable.

To overcome the drawbacks in the traditional solid-state reaction method, the chemical vapor deposition (CVD) process was deliberately developed to prepare Sr_2_Si_5_N_8_-based phosphors in the present research. Metallic strontium was used as a vapor source for producing Sr_3_N_2_ vapor to react with other raw materials during the reaction process. Because of the gas-solid reaction rather than the conventional liquid-solid reaction, the CVD process is beneficial for increasing the homogeneity of reaction and improving the quality of phosphors. For producing yellow-light emission, Ce^3+^ ions were doped into Sr_2_Si_5_N_8_ host. The phosphors prepared using the proposed CVD and conventional solid-state reaction processes were compared with regard to crystallinity, particle morphology and luminescence properties. The yellow emitting Sr_2_Si_5_N_8_:Ce^3+^ and commercial red emitting Sr_2_Si_5_N_8_:Eu^2+^ phosphors were then combined with blue LED chips for the fabrication of WLEDs to demonstrate the industrial application of the CVD-derived phosphors. The conceptual mechanism of the CVD process for the synthesis of yellow-emitting Sr_2_Si_5_N_8_:Ce^3+^ phosphors and the relevant characteristics may open up a new path for the advancement of lighting industry.

## Results and Discussions

### Phase identification and structure of Ce^3+^-doped Sr_2_Si_5_N_8_

**Figure 1:**
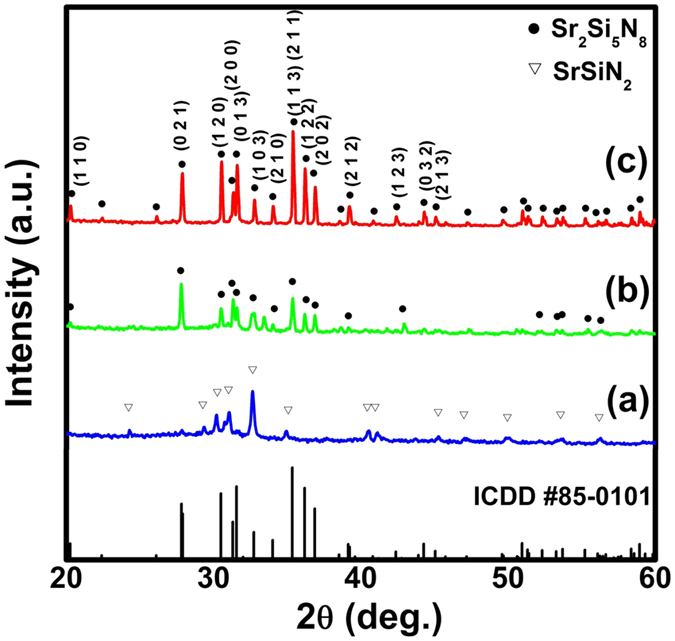
XRD patterns of Sr_1.94_Ce_0.06_Si_5_N_8_ phosphors synthesized via the CVD process at (**a**) 1400 °C, (**b**) 1500 °C, and (**c**) 1600 °C.

Sr_2−x_Ce_x_Si_5_N_8_ (x = 0.02–0.10) phosphors were prepared via the CVD process (method A). Figure 1 display XRD patterns of Sr_1.94_Ce_0.06_Si_5_N_8_ synthesized at various temperatures ranging from 1400 °C to 1600 °C. After annealing the precursors at 1400 °C, the compound of SrSiN_2_ was found to form. When the calcination temperature was increased from 1400 °C to 1500 °C, Sr_2_Si_5_N_8_ structure with low crystallinity was found without any trace of SrSiN_2_. Further increasing the annealing temperature to 1600 °C resulted in the formation of the Sr_2_Si_5_N_8_ structure with pure phase and high crystallinity. The recorded diffraction patterns of Sr_1.94_Ce_0.06_Si_5_N_8_ matched well with the standard pattern (ICDD No. 85-0101).

**Figure 2:**
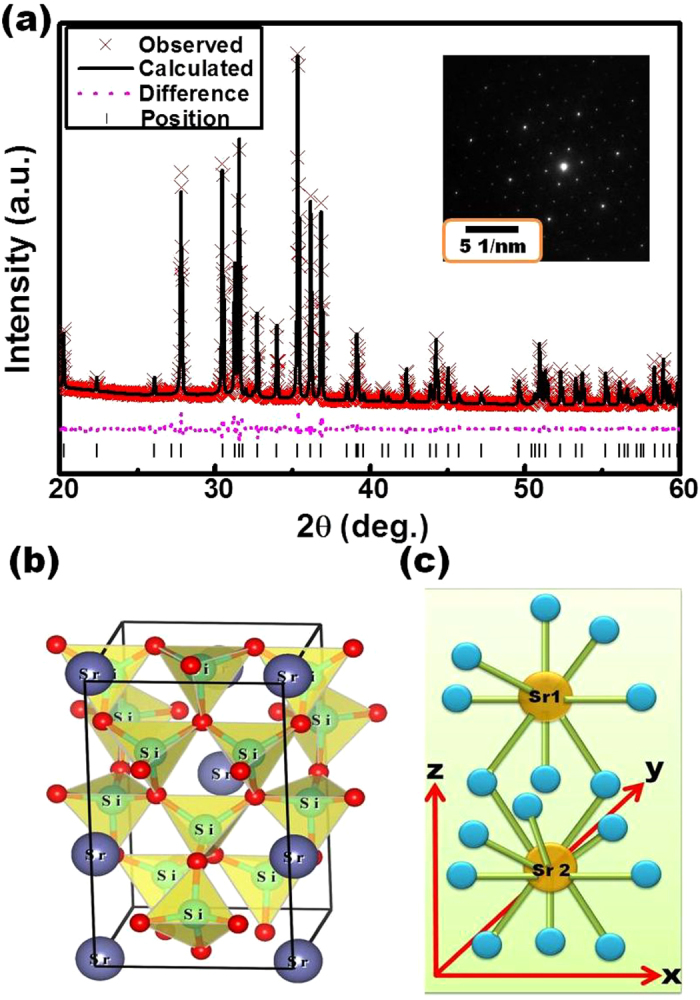
(**a**) Refinement pattern of observed (×) and calculated (solid line) X-ray diffraction patterns, difference profile (dot line), and positions of all the reflections (vertical bars) for Sr_1.94_Ce_0.06_Si_5_N_8_ phosphors prepared via the CVD process at 1600 °C. Inset: SAED pattern of Sr_1.94_Ce_0.06_Si_5_N_8_ phosphors. (**b**) Structural representation and (**c**) coordination environment of Sr^2+^ sites for Sr_1.94_Ce_0.06_Si_5_N_8_ phosphors.

Figure 2(a) presents the rietveld XRD pattern of Sr_1.94_Ce_0.06_Si_5_N_8_ synthesized via the CVD method at 1600 °C. The solid curve indicates the simulated diffraction data, the “×” marks represent the experimental diffraction data, the straight bars indicate the positions of simulated diffraction patterns, and the dotted line denotes the deviation between the simulated and experimental values. The calculated R_p_ and wR_p_ parameters were converged to reliable values of 0.0481 and 0.0643, respectively. The refinement results confirmed that Sr_1.94_Ce_0.06_Si_5_N_8_ belongs to the orthorhombic crystal system and the space group of Pmn2_1_ (no. 176). Table 1 lists the as-estimated lattice parameters of Sr_1.94_Ce_0.06_Si_5_N_8_. The calculated lattice parameters were a = 5.7100 Å, b = 6.8202 Å and c = 9.3349 Å, and the crystal size was 109.3 nm. The inset of Fig. 2(a) displays the SAED pattern of Sr_1.94_Ce_0.06_Si_5_N_8_ measured from HRTEM. The lattice plane (013) of Sr_2_Si_5_N_8_ can be identified from the diffraction spots in the SAED pattern. The sharp diffraction spots indicate the high crystallinity of the as-prepared phosphors.

From the refinement parameters, the crystal structure of Sr_1.94_Ce_0.06_Si_5_N_8_ was drawn via the VESTA software and shown in Fig. 2(b)^19^. From this figure, it is shown that Sr^2+^ ions are assembled in the channels formed via Si_6_N_6_ rings along the [100] orientation. Figure 2(c) indicates that there are two kinds of Sr^2+^ sites, Sr1 and Sr2 with the coordination numbers of 8 and 10, respectively. Both Sr^2+^ sites are occupied by Ce^3+^ ions. Furthermore, the mean distance of Sr1–N (2.865 Å) is smaller than that of Sr2–N (2.928 Å). Therefore, Ce^3+^ ions locating at the Sr1 sites is considered to experience stronger crystal field strength than those occupying the Sr2 site. The different crystal field strength between the two sites cause different luminescent properties, as shown in the later section.

**Figure 3:**
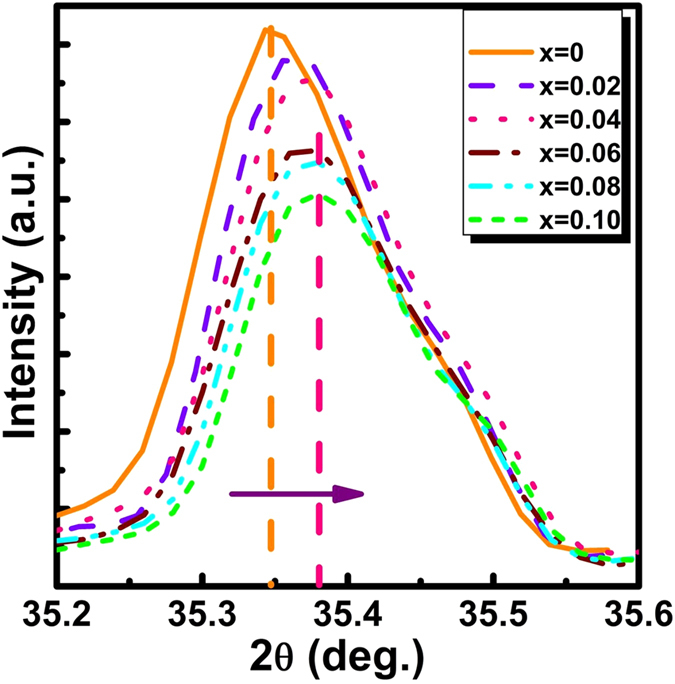
Variation of (113) XRD diffraction peak with the concentration of Ce^3+^ ions in Sr_2−x_Ce_x_Si_5_N_8_ phosphors.

As the doping concentration of Ce^3+^ ions in Sr_2−x_Ce_x_Si_5_N_8_ phosphors was increased from x = 0 to x = 0.10, all XRD patterns were identified to be Sr_2_Si_5_N_8_ phase without any impurity phases. A small peak shift was observed with the doping of Ce^3+^ ions. Figure 3 shows the shift of the XRD peak for Sr_2−x_Ce_x_Si_5_N_8_ (x = 0.02–0.10). As the doping of Ce^3+^ ions increased, the (113) peak shifted to high diffraction angles. The ionic radius of Ce^3+^ ion (115 pm) is smaller than that of Sr^2+^ ion (132 pm). Therefore, the lattice parameters of Sr_2_Si_5_N_8_ tended to decrease with the doping of Ce^3+^ ions, resulting in the shift of XRD peaks to high diffraction angles^20^.

### Comparison of crystal structures, morphology and luminescent properties between Ce^3+^-doped Sr_2_Si_5_N_8_ synthesized via the CVD and solid-state reaction processes

**Figure 4:**
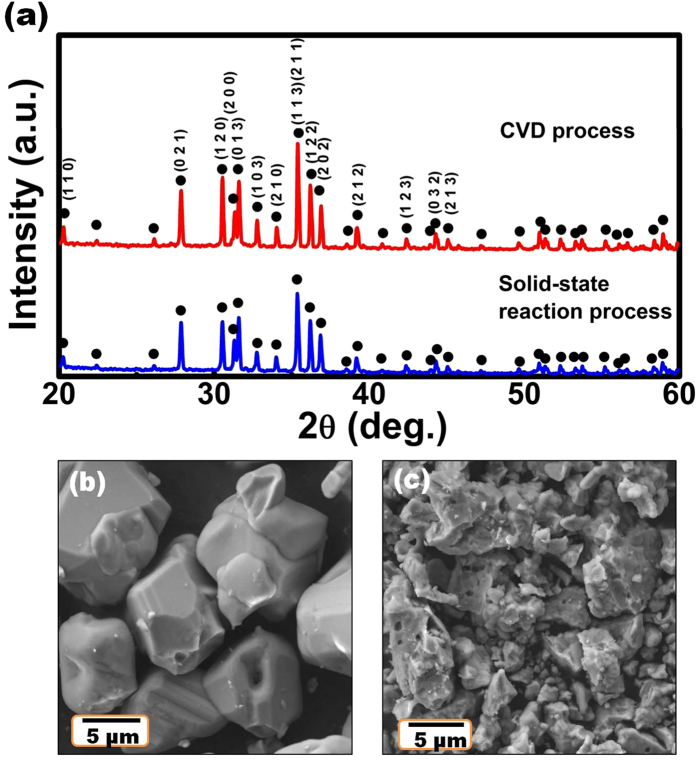
(**a**) A comparison for XRD patterns of Sr_1.94_Ce_0.06_Si_5_N_8_ phosphors synthesized via the CVD and solid-state reaction processes at 1600 °C. Scanning electron micrographs of Sr_1.94_Ce_0.06_Si_5_N_8_ phosphors prepared via the (**b**) CVD and (**c**) solid-state reaction processes.

Figure 4(a) displays the comparative XRD patterns for Sr_1.94_Ce_0.06_Si_5_N_8_ phosphors synthesized via methods A and B at 1600 °C. It was shown that both methods produced pure Sr_1.94_Ce_0.06_Si_5_N_8_ compound without any impurity. However, the diffraction peak intensity of phosphors prepared via method A is higher than that of phosphors prepared via method B. Figure 4(b,c) shows the scanning electron micrographs of Sr_1.94_Ce_0.06_Si_5_N_8_ phosphors synthesized via both methods. The phosphors prepared via the method A exhibited a size distribution in the range of 8–10 μm, as shown in Fig. 4(b). In contrast, the particle size of phosphors prepared via method B ranged from 0.5 μm to 5 μm, as shown in Fig. 4(c). These results indicated that phosphors prepared via method A exhibited larger particle size and smaller size distribution than those prepared via the method B.

**Figure 5:**
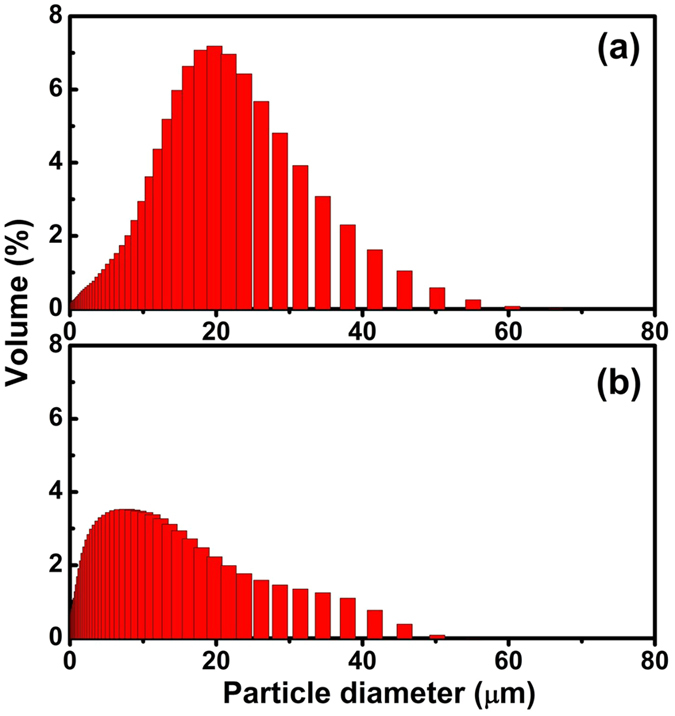
A comparison for particle size distribution of Sr_1.94_Ce_0.06_Si_5_N_8_ phosphors synthesized via the (**a**) CVD and (**b**) solid-state reaction processes at 1600 °C.

Figure 5(a,b) shows the particle size distribution of Sr_1.94_Ce_0.06_Si_5_N_8_ phosphors analyzed using the laser diffraction particle size analyzer. The average particle size of phosphors prepared via method A was 21.7 μm with a standard deviation of 8.7 μm. However, the average particle size of phosphors synthesized via method B was measured to be 9.7 μm with a standard deviation of 9.1 μm. Both the results of SEM and particle size analysis indicated that the CVD process was beneficial to prepare phosphors with large particle sizes and small size distribution.

**Figure 6:**
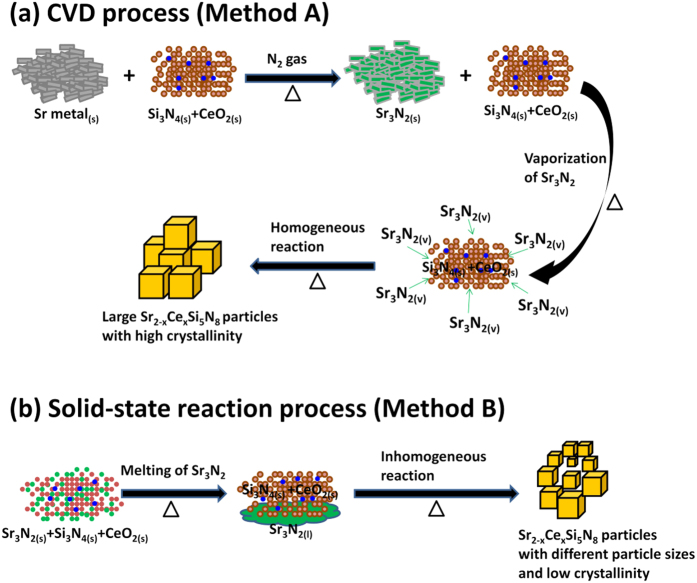
Reaction mechanism of the formation for Sr_2−x_Ce_x_Si_5_N_8_ phosphors via the (**a**) CVD and (**b**) solid-state reaction processes.

The reaction mechanism of the formation for Sr_2_Si_5_N_8_ phosphors via methods A and B are proposed in Fig. 6(a,b), respectively. In method A, Sr metal and the mixtures of Si_3_N_4_ and CeO_2_ are placed separately in two crucibles. During the heating process, Sr metal first reacts with N_2_ gas to form Sr_3_N_2_^21^. Then Sr_3_N_2_ melts to be liquid and produces Sr_3_N_2_ vapor at temperatures above the melting point of Sr_3_N_2_ (m.p. = 1030 °C). Sr_3_N_2_ vapor then flows with the carrier gas to react with the mixtures in the other crucible to form Sr_2−x_Ce_x_Si_5_N_8_. Owing to the high uniformity of gas-solid mixtures for Sr_3_N_2(v)_, Si_3_N_4(s)_, and CeO_2(s)_, the reaction occurs homogeneously. Therefore, the formed Sr_2−x_Ce_x_Si_5_N_8_ particles exhibited small size distribution. Furthermore, the high mobility of gas may enhance the diffusion process and the corresponding reaction rates, thereby resulting in the formation of large particles^22^,^23^. On the other hand, in method B, the mixtures of Sr_3_N_2_, Si_3_N_4_ and CeO_2_ are placed in the same crucible. When the temperature is increased over 1030 °C, Sr_3_N_2_ melts and flows to the bottom of the mixtures for Si_3_N_4_ and CeO_2_ powders. As a result, the reaction on the top of powders may be incomplete. The incomplete reaction will result in low crystallinity and weak XRD peak intensity^24^,^25^. In addition, the liquid-solid contact between Sr_3_N_2(l)_ and the mixtures of Si_3_N_4(s)_ and CeO_2(s)_ is heterogeneous and the reaction is inhomogeneous. Therefore, the formed Sr_2_Si_5_N_8_ particles show non-uniform size distribution.

**Figure 7:**
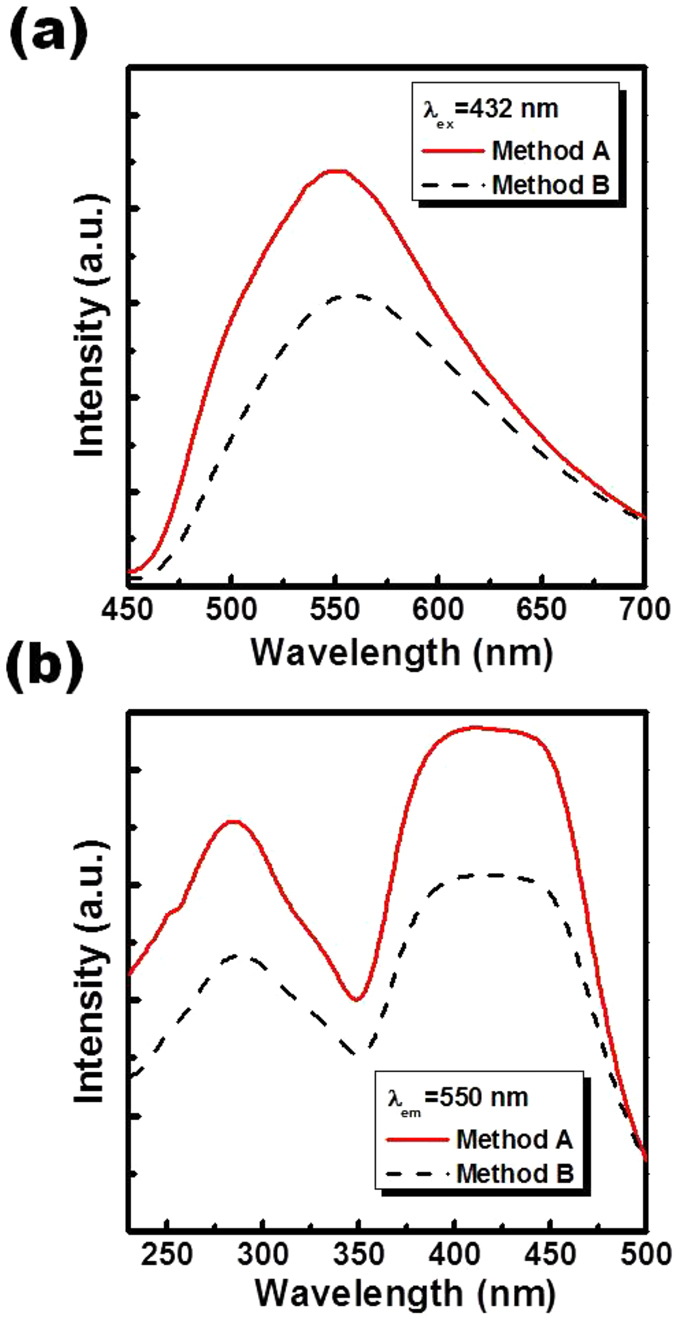
(**a**) Photoluminescence emission spectra and (**b**) excitation spectra of Sr_1.94_Ce_0.06_Si_5_N_8_ phosphors prepared via the CVD and solid-state reaction processes at 1600 °C.

Figure 7(a) presents the PL emission spectra of Sr_1.94_Ce_0.06_Si_5_N_8_ phosphors synthesized via methods A and B at 1600 °C. Under the blue excitation at 432 nm, Sr_1.94_Ce_0.06_Si_5_N_8_ prepared via both methods exhibited a broad emission band centered at approximately 550 nm due to the 5d-4f transition of Ce^3+^ ions^26^. The emission intensity of phosphors prepared via method A was approximately 40% higher than that prepared via method B. The enhanced emission properties of phosphors synthesized via method A than that synthesized via method B can be attributed to the high crystallinity of the prepared phosphors, as shown in Fig. 4(a)^27^. Moreover, it is known that small particles usually possess more surface defects than large particles and these surface defects may decrease the photoluminescence intensity of phosphors^28^,^29^,^30^. Therefore, the small particle size of the phosphors synthesized via method B (as shown in Fig. 4(c)) may also be the reason for the relative low photoluminescence intensity. Figure 7(b) displays the excitation spectra of Sr_1.94_Ce_0.06_Si_5_N_8_ phosphors synthesized via both methods at 1600 °C. The excitation spectra of phosphors prepared via both methods monitored at 550 nm were similar, and both spectra included two broad excitation bands at 230–350 nm and 350–500 nm, respectively. The peak at 285 nm can be attributed to the host lattice excitation, while the broad excitation band from 350 to 500 nm is due to the complex splitting of the 5d^1^ excited state (4f-5d transition) for Ce^3+^ ions^31^,^32^.

### Photoluminescence characteristics of Ce^3+^-doped Sr_2_Si_5_N_8_ host

**Figure 8:**
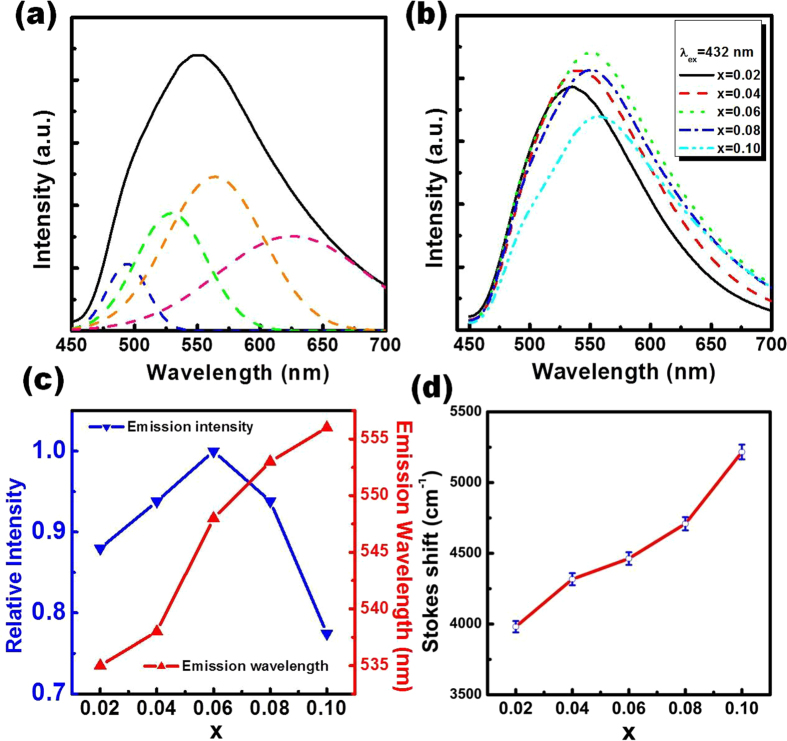
(**a**) Deconvoluted emission spectra of Sr_1.94_Ce_0.06_Si_5_N_8_, (**b**) emission spectra of Sr_2−x_Ce_x_Si_5_N_8_ (x = 0.02–0.10) phosphors synthesized via the CVD process at 1600 °C, and variation of the (**c**) emission intensity, peak wavelength, and (**d**) Stokes shift with the concentration of Ce^3+^ ions in Sr_2−x_Ce_x_Si_5_N_8_.

It was reported that Ce^3+^ ions in different host materials show two characteristics emission bands due to the spin-orbit splitting of the ground state (^2^F_5/2_ and ^2^F_7/2_) with an energy difference of approximately 2000 cm^−1^ 33. To further investigate the broad emission band of Ce^3+^ doped Sr_2_Si_5_N_8_, the emission curve was fitted to be four well-separated Gaussian components peaking at approximately 493 nm, 530 nm, 562 nm and 626 nm, as shown in Fig. 8(a). The energy difference between the sub-bands 493 nm and 530 nm was 1416 cm^−1^, and that between the sub-bands 562 nm and 626 nm was 1819 cm^−1^. These two values are close to the energy difference of the two ground states ^2^F_5/2_ and ^2^F_7/2_. Therefore, it can be concluded that Ce^3+^ ions are located at two different Sr^2+^ sites and eventually two kinds of luminescent centers are formed. S. Miao *et al*. suggested that the local environment surrounding Ce^3+^ ions in the host lattice can affect the positions of the emission band for Ce^3+^ ions and the positions can be estimated via an empirical relation given as follows^34^:


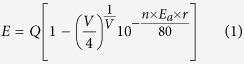


where E (cm^−1^) represents the real position of the d-band edge, Q represents the position in energy for the d-band edge of Ce^3+^ free ions, r is the radius of the host cation, and E_a_ is the electron affinity of the atoms. The valence of Ce^3+^ ions and the number of anions in the immediate shell around the ions are denoted by V and n, respectively. In the present case, Q, V, r and E_a_ are constants. Therefore, the positions of the emission band only depend on the number of anions in the immediate shell around three crystallographically independent cation sites in Sr_2_Si_5_N_8_matrix including Si^4+^, 8 coordinated Sr^2+^ and 10 coordinated Sr^2+^ sites. As per the estimation done in the literature, these three conditions were calculated based on Eq. (1), respectively^35^. Compared with the calculated values obtained from Si^4+^ sites (1200–1250 nm), it can be concluded that the Sr^2+^ sites can act as the favorable cation sites for Ce^3+^ ion. According to Eq. (1), Ce^3+^ ions locating at the 8-coordinate Sr^2+^ sites tend to exhibit longer emission wavelength (560–630 nm) than those occupying the 10-coordinate Sr^2+^ sites (489–535 nm). Therefore, it is considered that Ce^3+^ ions with the emissions at 493 nm and 530 nm occupies the 10-coordinate Sr^2+^ sites, while the other Ce^3+^ ions showing emissions at 562 nm and 626 nm are related to the 8-coordinate Sr^2+^ sites.

Figure 8(b) shows the emission spectra of Sr_2−x_Ce_x_Si_5_N_8_ (x = 0.02–0.10) phosphors calcined via method A at 1600 °C. The relationship between x and the relative emission intensity is shown in Fig. 8(c). Increasing the doping amount of Ce^3+^ ions to x = 0.06 led to an increase in the emission intensity of Sr_2−x_Ce_x_Si_5_N_8_. However, a further increase in the concentration of Ce^3+^ ions reduced the emission intensity due to the self concentration quenching phenomena^36^. The self-concentration quenching effects between two Ce^3+^ ions may be owing to the non-radiative energy transfer between two Ce^3+^ ions. In general, the non-radiative energy transfer between two identical ions may take place via exchange interaction, radiation reabsorption, or an electric multipolar interaction. With an increase in the concentration of identical ions, the distance between two ions reduces and the energy transfer starts at a critical distance (R_c_)^37^. R_c_ can be calculated from the structural parameters including cell volume (V), the number of cations in the unit cell (N), and the critical concentration of Ce^3+^ (C) in the host via the following formula^38^:


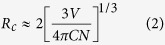


From the appropriate V, N and C values (363.53 Å^3^, 2 and 0.06, respectively), R_c_ in Sr_2−x_Ce_x_Si_5_N_8_ was calculated to be 17.95 Å. According to G. Blasse, R_c_ for the general exchange interaction is estimated to be around 5 Ǻ^38^. Therefore, the exchange interaction can be neglected in the energy transfer within Sr_2−x_Ce_x_Si_5_N_8_ phosphors. In the present case, radiation reabsorption and multipolar interaction may be the main mechanism for concentration quenching. On the other hand, when x was increased from 0.02 to 0.10, the emission peak position for phosphors shifted from 535 nm to 556 nm. Such resultant shift may be due to the re-absorption of high energy emission and the increased Stokes shift owing to the change of crystal field strength^39^. Figure 8(d) shows the Ce^3+^ concentration dependent Stokes shift estimated from the energy difference between the last excitation band at low energy and the first emission band at high energy^14^. It can be seen clearly that the Stokes shift increased with increasing Ce^3+^ concentrations and hence supported the shifting of emission peak position.

### Quantum efficiency and thermal stability of Ce^3+^-doped Sr_2_Si_5_N_8_ host

**Figure 9:**
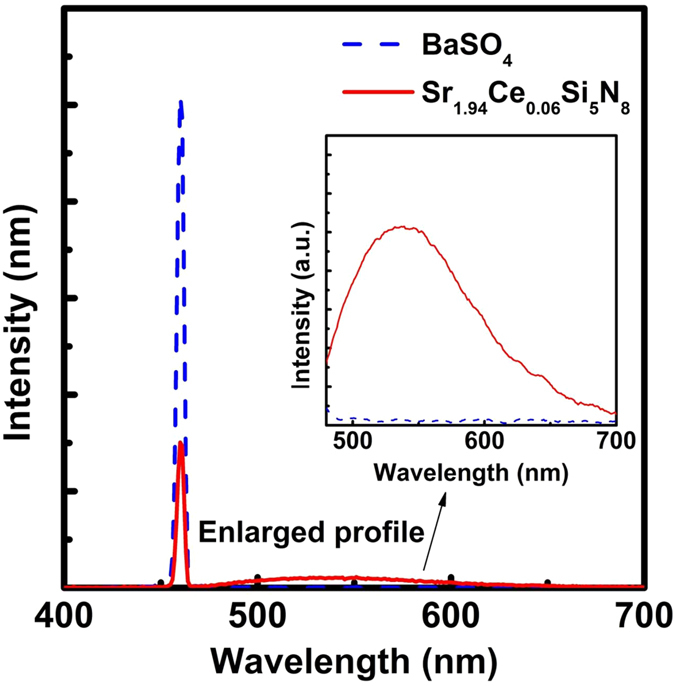
Luminescence spectra of BaSO_4_ powders and Sr_1.94_Ce_0.06_Si_5_N_8_ phosphors collected from an integrating sphere under excitation at 460 nm.

The integrating sphere was applied for determining the absolute quantum efficiency of the as-prepared phosphors. BaSO_4_ powders with a reflectivity of 95% in visible light were used as the standard to calculate the spectrum of the excitation source. Figure 9 shows the luminescence spectra of BaSO_4_ powders and Sr_1.94_Ce_0.06_Si_5_N_8_ phosphors under the excitation at 460 nm. The external quantum efficiency (EQE) can be calculated using the formula η_o_ = N_em_/N_exc_ × 100%, where N_em_ and N_exc_ are the number of emitted and excited photons, respectively. The internal quantum efficiency (IQE) can be calculated from η_i_ = N_em_/N_abs_ × 100%, where N_abs_ is the number of absorbed photons^40^. The EQE and IQE for Sr_1.94_Ce_0.06_Si_5_N_8_ phosphors were calculated to be 51% and 71%, respectively. These results indicate that Sr_1.94_Ce_0.06_Si_5_N_8_ phosphors have potential to be used in 460 nm InGaN-based LED chips.

**Figure 10:**
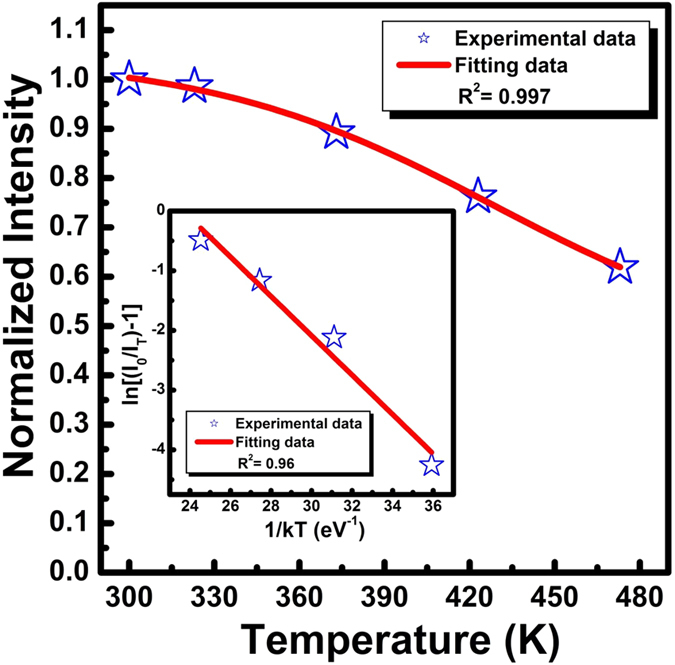
Photoluminescence intensity for Sr_1.94_Ce_0.06_Si_5_N_8_ phosphors as a function of temperatures. Inset: plot of ln[(I_0_/I_T_) − 1] vs 1/kT for the phosphors.

Figure 10 plots the normalized emission intensity of Sr_1.94_Ce_0.06_Si_5_N_8_ phosphor as a function of temperature under the blue excitation at 460 nm. The photoluminescence intensity was observed to decrease with the increase in temperature. At 150 °C, the emission intensity remained approximately 73% of that recorded at room temperature. The decrease in emission intensity was fit with Boltzmann sigmoidal function properly with R^2^ value larger than 0.99 and the fitting were employed in the estimation of TQ_1/2_ value. TQ_1/2_ is the temperature at which the phosphor loses half of its emission efficiency. From the fitting data, TQ_1/2_ was obtained as a high value of 513 K (±9 K). When the temperature was increased, the nonradiative relaxation probability induced by enhanced phonon-electron interaction was also increased^41^. The activation energy (*E*_*a*_) for thermal quenching can be obtained using the equation listed below^42^,^43^:


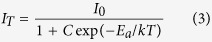


where I_0_ and I_T_ are the luminescence intensities at room and testing temperatures, respectively, C is a constant, and k is the Boltzmann constant (8.617 × 10^−5^ eV K^−1^). The inset in Fig. 10 plots ln[(I_0_/I_T_) − 1] vs. 1/kT to calculate the activation energy for Sr_1.94_Ce_0.06_Si_5_N_8_. From Eq. (3), E_a_ was estimated to be 0.33 eV (±0.04 eV). The high value of activation energy indicates high thermal stability for the present phosphors. Such high thermal stability due to the compact crystal lattice of Sr_2_Si_5_N_8_-based structure is suitable for LED applications.

### Electroluminescence properties of phosphors-converted white LEDs

**Figure 11:**
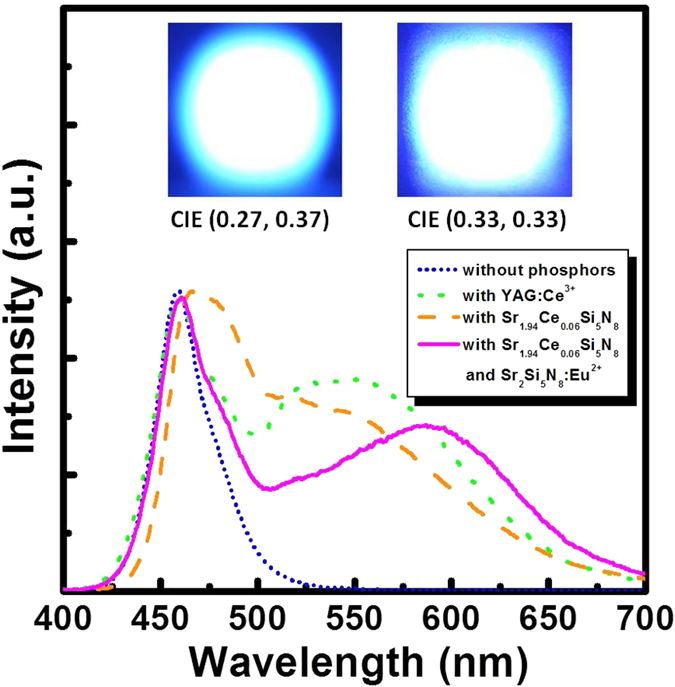
EL spectra of YAG:Ce^3+^, Sr_1.94_Ce_0.06_Si_5_N_8_ and Sr_2_Si_5_N_8_:Eu^2+^ phosphors coated 460 nm blue LED chips. Photos: the images of packaged LEDs driven by a current of 280 mA.

Herein, an effective synthesis route has been designed to produce high quality Sr_2_Si_5_N_8_:Ce^3+^ nitridosilicate phosphors for LEDs. The developed phosphors synthesized via the novel CVD route showed high yellow-emission intensity, adequate quantum efficiency, and very low thermal quenching behaviors. The research outcomes directly indicate the suitability of the present phosphors for possible LED applications. Therefore, the optimum composition derived from the CVD route was finally incorporated in LED packaging to check the suitability of the phosphors for industrial applications. Figure 11 shows the electroluminescence (EL) spectra of LEDs driven by a current of 280 mA. When Sr_1.94_Ce_0.06_Si_5_N_8_ phosphor was applied to a LED chip, the electroluminescence spectrum presented a blue peak at 460 nm as well as the yellow emission band of Sr_1.94_Ce_0.06_Si_5_N_8_. The corresponding CIE coordinate was (0.27, 0.37) with a near white CCT of 8167 K and a R_a_ value of 64. On the other hand, the conventional YAG:Ce^3+^ phosphors were also packed with blue LEDs having a 460-nm emission. The corresponding CIE coordinate of YAG:Ce^3+^-coated LEDs was (0.29, 0.36) with a CCE of 7161 K and a R_a_ value of 69. Both two kinds of LEDs showed low CRI values owing to the lack of red emissions. The high CCT result indicates that Sr_1.94_Ce_0.06_Si_5_N_8_-coated LED exhibits the color temperature in the cold white region^43^. For improving the CCT and CRI of LEDs, Sr_2_Si_5_N_8_:Eu^2+^ phosphors were blended with Sr_1.94_Ce_0.06_Si_5_N_8_ and coated on another LED chip. The resulting electroluminescence spectrum exhibited a combination of blue, yellow, and red emissions with corresponding CIE coordinates of (0.33, 0.33), a pure white CCT of 5953 K, and R_a_ of 84 which is close to standard daylight at noon (D65, 6500 K) and can be applied for different commercial appliances^44^. Table 2 lists the full set of CRI and average CRI (R_a_) values. The insets of Fig. 11 present the images of the packaged WLEDs. The light emission with high brightness was seen clearly. As a consequence of the present work, yellow emission Sr_2−x_Ce_x_Si_5_N_8_ with high crystallinity and high brightness were successfully synthesized via the CVD process. The potential of the present phosphors for application in WLEDs was demonstrated.

### Conclusions

A chemical vapor deposition (CVD) process was newly developed to synthesize Sr_2_Si_5_N_8_: Ce^3+^ phosphors through the reaction between Sr_3_N_2(v)_ and the mixtures of Si_3_N_4(s)_ and CeO_2(s)_. The phosphors prepared via the CVD process had high crystallinity, uniform particle size distribution in the range of 8–10 μm and efficient photoluminescence due to the homogeneous gas-solid reaction. On the other hand, the phosphors prepared via the solid-state reaction process showed low crystallinity, nonuniform size distribution in the range of 0.5–5 μm, relatively low photoluminescence because of the inhomogeneous liquid-solid reaction. As the concentration of Ce^3+^ ions in Sr_2−x_Ce_x_Si_5_N_8_ was increased from x = 0.02 to 0.10, a red shift of the emission peak from 535 nm to 556 nm was observed under blue light excitation. Meanwhile, phosphors exhibited the maximum emission intensity at x = 0.06. The critical distance (R_c_) of energy transfer, the external and internal quantum efficiencies were calculated to be 17.95 Å, 51% and 71%, respectively. The activation energy of thermal stability for Sr_2_Si_5_N_8_:Ce^3+^ was counted to be 0.33 eV. A white LED with a color rendering index of 84 and a color temperature of 5953 K was fabricated via utilizing the mixture of Sr_2_Si_5_N_8_:Ce^3+^ and Sr_2_Si_5_N_8_:Eu^2+^ phosphors with a InGaN LED chip (460 nm). This research demonstrated a potential synthesis technique to prepare nitride phosphors for white LEDs.

## Materials and Methods

### Synthesis of Ce^3+^-doped Sr_2_Si_5_N_8_ phosphors via the CVD process

**Figure 12:**
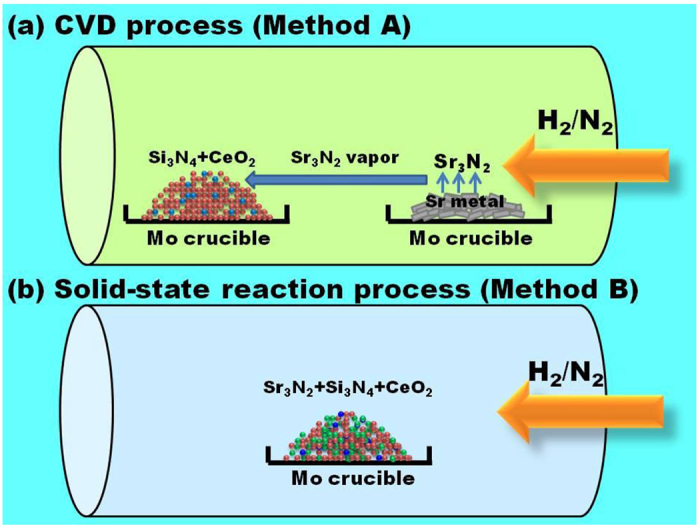
Schematic diagrams for forming Sr_2−x_Ce_x_Si_5_N_8_ phosphors via the (**a**) CVD and (**b**) solid-state reaction processes.

In the present work, Sr_2−x_Ce_x_Si_5_N_8_ phosphors were prepared via the chemical vapor deposition (CVD) process. Figure 12(a) illustrates the schematic diagram of the CVD process, which is defined as method A. 0.012 mol of strontium metal was put in a molybdenum crucible. The mixtures of analytical-grade Si_3_N_4_ (0.005 mol) and CeO_2_ (0.003x mol, x = 0.02–0.10) powders were put in another molybdenum crucible. Then both molybdenum crucibles were placed in a tubular furnace for heating under a H_2_/N_2_ mixed atmosphere. The partial pressures of H_2_ and N_2_ were 76 torr H_2_ and 684 torr, respectively. The annealing temperature was increased to 800 °C and maintained for 1 h to nitridize strontium metal to be strontium nitride. Then the heating temperatures were further increased to 1400–1600 °C and maintained for 8 h to evaporate strontium nitride onto the mixture powders to form Sr_2−x_Ce_x_Si_5_N_8_ (x = 0.02–0.10) phosphors through the CVD process.

### Synthesis of Ce^3+^-doped Sr_2_Si_5_N_8_ phosphors via the solid-state reaction process

In order to compare the phosphors prepared via the CVD process with those prepared via the conventional process, Sr_1.94_Ce_0.06_Si_5_N_8_ phosphors were also synthesized via the conventional solid-state reaction process. Figure 12(b) illustrates the schematic diagram of the solid-state reaction process, which is defined as method B. Analytical-grade Sr_3_N_2_ (0.00194 mol), Si_3_N_4_ (0.005 mol) and CeO_2_ (0.00018 mol) powders were thoroughly ground and mixed according to the chemical formula Sr_1.94_Ce_0.06_Si_5_N_8_ in an argon-filled glove box. Then the mixed powders were placed in a molybdenum crucible and calcined at 1600 °C for 8 h in a reduced atmosphere (76 torr H_2_ and 684 torr N_2_) to form Sr_1.94_Ce_0.06_Si_5_N_8_ phosphors.

### Characterization of phosphors

The structural analysis of the obtained samples was carried out using X-ray diffractometer (Rigaku, Ultima IV) with a standard *CuK*_*α*_ X-ray source. The PDXL program was used to refine the structure. The microstructures of the prepared phosphors were performed using a field emission scanning electron microscope (FE-SEM) (JEOL JSM-7600F) and a field emission transmission microscope (FE-TEM) (Philips Tecnai F30). The particle size distribution of phosphors was carried out using a laser diffraction particle size analyzer (Coulter, LS230). The photoluminescence characteristics of the prepared phosphors were investigated using a fluorescence spectrophotometer (Hitachi, F-4500) with a Xe lamp as the excitation source. The quantum efficiency was measured using a CCE spectrophotometer (BRC112E) with an integrating sphere. The thermal stability of the as-prepared phosphors was measured using a CCE spectrophotometer (BRC112E) and a heater.

### Fabrication and characterization of WLEDs

For fabricating white LEDs, the as-prepared phosphors were mixed with commercial Sr_2_Si_5_N_8_:Eu^2+^ phosphors and dispersed in transparent silicon resin to prepare phosphor mixtures. The mixtures were then coated on 460 nm InGaN-based LED chips to fabricate LED devices. The photoluminescence characteristics of fabricated LEDs were measured using a CCE spectrophotometer (BRC112E). The Commission Internationale de I’Eclairage (CIE) coordinates were converted from the photoluminescence spectra using the color calculator software.

## Additional Information

**How to cite this article**: Yang, C.-Y. *et al*. Synthesis of Sr_2_Si_5_N_8_:Ce^3+^ phosphors for white LEDs via an efficient chemical deposition. *Sci. Rep.* **7**, 45832; doi: 10.1038/srep45832 (2017).

**Publisher's note:** Springer Nature remains neutral with regard to jurisdictional claims in published maps and institutional affiliations.

## Figures and Tables

**Table 1:** Crystal structural data and lattice parameters of Sr_1.94_Ce_0.06_Si_5_N_8_ synthesized via the CVD process at 1600 °C.

Process	CVD
Crystal system	Orthorhombic
Space group	Pmn2_1_
Lattice constants	
a (Å)	5.7100
b (Å)	6.8202
c (Å)	9.3349
α (deg.)	90
β (deg.)	90
γ (deg.)	90
Crystal size (nm)	109.3
R values	ωR_p_ = 0.0643
	R_p_ = 0.0481

**Table 2:** Full set of 15 CRIs and R_a_ for 460 nm blue chips with YAG:Ce^3+^, Sr_1.94_Ce_0.06_Si_5_N_8_ and Sr_2_Si_5_N_8_:Eu^2+^ phosphors.

	R_1_	R_2_	R_3_	R_4_	R_5_	R_6_	R_7_	R_8_	R_9_	R_10_	R_11_	R_12_	R_13_	R_14_	R_15_	R_a_
Blue LEDs with YAG:Ce^3+^	61	82	89	57	65	79	80	45	−77	63	54	49	66	92	48	69
Blue LEDs with Sr_1.94_Ce_0.06_Si_5_N_8_	60	83	72	43	64	85	67	41	−73	72	43	56	65	83	45	64
Blue LEDs with Sr_1.94_Ce_0.06_Si_5_N_8_ and S_2_Si_5_N_8_:Eu^2+^	91	94	85	77	88	89	79	70	34	92	77	70	97	93	89	84
