# Corrigendum: Synthesis of Sr_2_Si_5_N_8_: Ce^3+^ phosphors for white LEDs via efficient chemical vapor deposition

**DOI:** 10.1038/srep46829

**Published:** 2017-07-11

**Authors:** Che-Yuan Yang, Sudipta Som, Subrata Das, Chung-Hsin Lu

Scientific Reports 7: Article number: 45832; 10.1038/srep45832 published online: 03 31 2017; updated: 07 11 2017.

The original version of this Article contained typographical errors in the title, where:

“Synthesis of Sr_2_Si_5_N_8_: Ce^3+^ phosphors for white LEDs via an efficient chemical deposition”.

now reads:

“Synthesis of Sr_2_Si_5_N_8_: Ce^3+^ phosphors for white LEDs via efficient chemical vapor deposition”.

This has now been corrected in the HTML and PDF versions of this Article.

